# Breast cancer with different prognostic characteristics developing in Danish women using hormone replacement therapy

**DOI:** 10.1038/sj.bjc.6601996

**Published:** 2004-07-06

**Authors:** C Stahlberg, A T Pedersen, Z J Andersen, N Keiding, Y A Hundrup, E B Obel, S Møller, F Rank, B Ottesen, E Lynge

**Affiliations:** 1 Department of Gynaecology and Obstetrics, Juliane Marie Centre, H:S Rigshospitalet, University of Copenhagen, Denmark; 2 Department of Biostatistics, University of Copenhagen, Denmark; 3 The Danish Nurse Cohort, The National Institute of Public Health, Copenhagen, Denmark; 4 DBCG-registry, Dep. 7003, H:S Rigshospitalet, Copenhagen, Denmark; 5 Centre of Diagnostic Investigations, Department of Pathology, H:S Rigshospitalet, University of Copenhagen, Denmark; 6 Institute of Public Health, University of Copenhagen, Denmark

**Keywords:** hormone replacement therapy, breast neoplasm, malignancy grade, receptor status, epidemiology, oestrogen, progestin

## Abstract

The aim of this study is to investigate the risk of developing prognostic different types of breast cancer in women using hormone replacement therapy (HRT). A total of 10 874 postmenopausal Danish Nurses were followed since 1993. Incident breast cancer cases and histopathological information were retrieved through the National Danish registries. The follow-up ended on 31 December 1999. Breast cancer developed in 244 women, of whom 172 were invasive ductal carcinomas. Compared to never users, current users of HRT had an increased risk of a hormone receptor-positive breast cancer, but a neutral risk of receptor-negative breast cancer, relative risk (RR) 3.29 (95% confidence interval (CI): 2.27–4.77) and RR 0.99 (95% CI: 0.42–2.36), respectively (*P* for difference=0.013). The risk of being diagnosed with low histological malignancy grade was higher than high malignancy grade with RR 4.13 (95% CI: 2.43–7.01) and RR 2.17 (95% CI: 1.42–3.30), respectively (*P*=0.063). For breast cancers with other prognostic characteristics, the risk was increased equally for the favourable and nonfavourable types. Current users of HRT experience a two- to four-fold increased risk of breast cancer with various prognostic characteristics, both the favourable and nonfavourable types. For receptor status, the risk with HRT was statistically significantly higher for hormone receptor-positive breast cancer compared to receptor-negative breast cancer.

Several studies have proposed that breast cancers diagnosed in women using hormone replacement therapy (HRT) may be smaller, with less spread to the regional lymph nodes (LN) and of lower histological malignancy grade, compared with those of never users. These findings could partly be explained by the biological influence of exogenous sex hormones on breast tissue as well as surveillance of women using HRT. The latest pooled analysis in 1997 concluded that breast cancers diagnosed in ever users of HRT were less likely to have spread to the regional lymph nodes or more distant sites compared to those developing in women never having used HRT. Furthermore, the excess risk of breast cancer in current or recent users was confined to localised disease. A total of 54% of the pooled studies contributed to this analysis (Collaborative Group on hormonal factors in breast cancer, 1997). Recent studies have added to the evidence, and found that use of HRT at the time of diagnosis is associated with one or more favourable prognostic characteristics, such as smaller tumour size, less regional lymph node involvement, lower tumour grade and lower stage of disease (Harding *et al*, 1996; Magnusson *et al*, 1996; Holli *et al*, 1997; Bilimoria *et al*, 1999; Ross *et al*, 2000; Esteve *et al*, 2002; Sacchini *et al*, 2002; Gertig *et al*, 2003; Kerlikowske *et al*, 2003). Other studies have not been able to detect such associations (Schairer *et al*, 2000; Stallard *et al*, 2000). Based on *in vivo* studies, it has been suggested that, while the effect of oestrogens is tumour promoting and carcinogenic, the effect of progestins on breast epithelium is both proliferative and inhibitory and that progesterone induces cellular differentiation (Thuneke *et al*, 2000; Alkhalaf *et al*, 2002; Lin *et al*, 2003). Breast carcinomas developing in premenopausal women and during or short after pregnancy, when endogenous levels of both oestrogens and progestins are high, have been found to be associated with poor histological and prognostic factors (Middleton *et al*, 2003; Reed *et al*, 2003).

Increased surveillance of women using HRT may contribute to the findings of apparently more favourable prognostic breast cancer types at the time of diagnosis and some studies find that adjusting for mammographic screening diminishes the magnitude of the effect (Daling *et al*, 2003). Results from the WHI trial in USA showed that breast cancers diagnosed in women randomised to HRT were of the same histology and grade as breast cancers diagnosed in the placebo group. However, with a difference of mean 2 mm, the tumours in HRT users were significantly larger and at a more advanced stage at the time of diagnosis, than tumours developing in the placebo group. Difficulties in detecting smaller tumours could be due to the higher percentage of increased mammographic density found in users of HRT, leading to delayed diagnosis (Chlebowski *et al*, 2003). Others have found that despite a lower probability of detecting early breast cancers in users of HRT at the time of mammographic examinations, the histological malignancy grade was lower in HRT users compared to never users (Esteve *et al*, 2002).

Only a few studies have analysed the different prognostic characteristics in breast cancer according to different HRT types and regimens (Magnusson *et al*, 1996; Ross *et al*, 2000; Schairer *et al*, 2000; Daling *et al*, 2003). Traditionally, HRT types and regimens have differed in the USA and some parts of Europe, especially in the Scandinavian countries. European studies have in general tended to report higher risk estimates of breast cancer in users of HRT, especially following the use of the continuous combined regimen with oestradiol and testosterone-like progestins. (Magnusson *et al*, 1999, 2000; Jernstrom *et al*, 2003; Olsson *et al*, 2003; Stahlberg *et al*, 2004). Recently, the Million Women Study from the UK found an increased risk of breast cancer, most pronounced for the combined oestrogen plus progestin treatment, but with no difference in risk estimates with respect to the cyclical or continuous treatment mode (Beral, 2003). The issue to be solved is whether different types and regimens of HRT influence the risk of developing breast cancer with various prognostic characteristics.

Most studies have analysed the distribution of the various prognostic factors of breast cancer according to the use of HRT, in women who have already developed the disease. This will not answer the question whether the risk of developing a favourable breast cancer is actually higher or lower than the risk of developing a non-favourable breast cancer for users of HRT compared to never users.

The aim of this study was to investigate how the current use of different HRT types and regimens at baseline influences the risk of breast cancers of both favourable and nonfavourable prognostic characteristics at the time of diagnosis, in a population of natural postmenopausal women without prior cancer diseases; furthermore, to assess if there is any difference between these risk estimates.

## MATERIALS AND METHODS

The setting of the Danish nurse cohort and the definition and characteristics of the study population has previously been described in detail (Stahlberg *et al*, 2004). Briefly, the Danish nurse cohort was established in 1993, when all Danish nurses above the age of 44 years received a mailed questionnaire (Hundrup *et al*, 2000). A total of 19 898 returned the questionnaire (86%).

### Ascertainment of exposure and possible confounders

The mailed questionnaire of 1993 served as baseline information. The questionnaire comprised detailed questions on the current use of HRT, including type, regimen and duration of use. Brand names were listed to facilitate the identification of HRT type. Furthermore, the questionnaire included details on known risk factors for breast cancer such as menarche, parity, age at first birth, alcohol use, physical activity, BMI, benign breast disease, oral contraceptives, hysterectomies performed, menopausal status and type of menopause.

### Ascertainment of end point

Breast cancer cases were identified by linkage to the Danish Cancer Registry (a registry with information on all cancer diagnoses since 1943), the Danish Breast Cancer Group Cooperation (DBCG) register (a clinical database on all breast cancer operations in Denmark including information on prognostic factors and histopathological details at the time of diagnosis since 1977) and the Lands patient Register (LPR) registry (the National Registry of Patients containing information on all hospital admissions, discharge diagnoses and operations performed since 1977). Self-reported information was evaluated together with the data derived from the registries. Information on tumour type, histological malignancy grade, hormone receptor status, axillary lymph status and stage of disease was obtained from the DBCG register. The follow-up started with the questionnaire in 1993, and ended on 31 December 1999.

### Analysis

The cohort comprised a total of 19 898 responders at baseline. We excluded all previously identified breast cancer cases, carcinoma *in situ* (CIS) of the breast and other invasive cancers, except for non-melanoma skin cancer (*n*=1086) at baseline. Furthermore, women with missing information on HRT (*n*=267), premenopausal women (*n*=5084) and women with a surgical menopause (*n*=571) were excluded. Also, hysterectomised women (*n*=2016) were excluded, as information on menopausal status and age at menopause would be missing in these women, which could seriously bias the analyses as described by Pike *et al* (1998). More than one exclusion criterion was fulfilled for several women. Finally, a total of 10 874 women were available for follow-up and analysis.

Women were considered postmenopausal if the menstrual bleeding had ceased or they were currently using HRT. The variable on menopausal age was constructed using the age at initiating HRT use or the cessation of menstrual bleeding, whichever event occurred first. The different HRT brands reported in the questionnaire were categorised according to the type and regimens used at baseline, that is, the systemic use of oestrogen only and oestrogen combined cyclically or continuously with progestin. Vaginal applied oestrogen users were considered non-users.

Data on prognostic characteristics were available from the DBCG Registry. The following variables were created: tumour size: ⩽2 cm/>2 cm, axillary lymph status: neg/pos, histological malignancy grade: grade 1/grade 2 and 3, oestrogen and progesterone receptor status: pos/neg, histology: invasive ductal/lobular/others. Information on staging was computed: stage according to TNM: stage1/stage 2–4. Nottingham Prognostic Index (NPI) was calculated as (tumour size (cm) × 0.2)+malignancy grade 1–3+lymph nodes 1–3 (1=neg, 2=1–3 pos LN, 3=more than 3 pos LN). The index was grouped into good prognosis (score<3.4)/moderate prognosis (score 3.5–5.4)/poor prognosis (score >5.4) (Haybittle *et al*, 1982). The analysis on prognostic characteristics was restricted to invasive ductal carcinomas.

### Statistical analysis

The Cox Proportional Hazards (PH) Model for left-truncated and right-censored data was used in the modelling of time-to-breast-cancer outcomes. The nurse's age was used as an underlying time with the age at study entry as the delayed entry time in the analysis. Women with histologies other than invasive ductal carcinomas were censored at the time of breast cancer diagnosis, as well as breast cancers with missing information on one of the prognostic characteristics. The various prognostic different breast cancer types were considered as single end points. Each prognostic factor was modelled in a multivariate Cox PH model, where relative risk (RR) and their 95% confidence intervals were estimated for HRT-exposure variable, adjusted for only significant risk factors (age at menopause, age at menarche, parity, age at first childbirth, use of oral contraceptives (OC), former benign breast disease (BBD), smoking, night work, body-mass index (BMI), height, physical activity, alcohol intake). Effect modification of HRT by menopausal age or by BBD was tested. Finally, for each of the prognostic factors, the RR estimates for the favourable and nonfavourable breast cancer outcomes in current HRT users were analysed in a competing risks framework using the Wald test. The likelihood ratio test for two competing risks as described in Rosthøj *et al* was additionally performed, confirming Wald test results (Andersen *et al*, 2002). The analysis was performed in Stata version 7.0.

## RESULTS

In this population of 10 874 natural postmenopausal women, a total of 2726 women (25.1%) were current users of HRT, 1582 women were past users (14.5%) and 6566 women (60.4%) were never users of HRT at baseline in 1993. Information on the type and regimen of HRT was available for current users.

A total of 244 women developed invasive breast cancer during the observation period from baseline in 1993 until 31 December 1999. Histological information was complete for 230 women: 172 invasive ductal carcinomas (74.8%), 36 lobular (15.6%) and 22 ‘other histologies’ such as mucinuous, medullary, tubullary and others (9.6%).

The analysis of breast cancer with different prognostic characteristics was restricted to women diagnosed with invasive ductal carcinomas (*n*=172). Receptor-positive breast cancer was seen in 79.8%, low histological malignancy grade was found in 37.3%, negative lymph node status in 56.4%, small tumour size in 58.1%, low stage in 40.1% and good prognostic group according to NPI in 39.5% (Table 1

**Table 1:** Descriptive statistics of prognostic factors in women with invasive ductal carcinomas (*n*=172)

**Variable**	**Categories**	**All frequency**
Receptor status	Receptor positive	126 (79.8%)
	Receptor negative	32 (20.2%)
	Missing^a^	14
		
Malignancy grade	Grade 1	60 (37.3%)
	Grade 2–3	101 (62.7%)
	Missing	11
		
Lymph node (LN) status	Node negative	93 (56.4%)
	Node positive	72 (43.6%)
	Missing^a^	7
		
Tumour size	2 cm or less	97 (58.1%)
	More than 2 cm	70 (41.9%)
	Missing^a^	5
		
Stage (TNM)	Stage 1	65 (40.1%)
	Stage 2–4	97 (59.9%)
	Missing^a^	10
		
Nottingham Prognostic Index (NPI)	Good prognosis	60 (39.5%)
	Moderate prognosis	77 (50.7%)
	Poor prognosis	15 (9.9%)
	Missing^a^	20

a Missing values are excluded from the analysis.

).

Overall, the risk of breast cancer of all types (*n*=244) was increased for current users of HRT, multivariate RR 2.42 (1.81–3.26) compared to never users of HRT. The risk of being diagnosed with invasive ductal carcinomas (*n*=172) was increased as well, multivariate RR 2.49 (1.76–3.51) compared to never users of HRT (Table 2

**Table 2:** The risk of breast cancer with all histologies and invasive ductal carcinoma following the use of HRT

**Use of HRT**	**No of HRT users**	**No of cases**	**Breast cancer all types**	**No of cases**	**Breast cancer invasive ductal carcinoma**
***N*=10 874**	***N* (%)**	***N*=244**	**RR (95% CI)^a^**	***N*=172**	**RR (95% CI)^a^**
Never	6566 (60.4)	110	1.00	78	1.00
Past	1582 (14.5)	31	1.16 (0.76–1.77)	16	0.85 (0.49–1.50)
Current	2726 (25.1)	103	2.42 (1.81–3.26)	78	2.49 (1.76–3.51)
Never HRT	6566 (60.4)	110	1.00	78	1.00
Past HRT	1582 (14.5)	31	1.16 (0.76–1.77)	16	0.85 (0.49–1.50)
Oestrogen	543 (5.0)	16	1.95 (1.15–3.32)	12	2.03 (1.10–3.75)
E+ prog-P cyclical	433 (4.0)	20	3.02 (1.80–5.05)	15	3.10 (1.69–5.67)
E+ test-P cyclical	1054 (9.7)	32	1.94 (1.26–3.00)	26	2.15 (1.31–3.54)
E+ test-P continuous	431 (3.1)	23	4.16 (2.56–6.75)	17	4.10 (2.29–7.30)
Current Tibolone	79 (0.7)	5	4.27 (1.74–10.51)	4	4.89 (1.78–13.42)
Current other HRT	276 (2.5)	7	1.53 (0.67–3.50)	4	1.10 (0.34–3.45)

E=oestrogen, P=progestin, prog-P=progesterone-like progestin, test-P=testosterone-like progestin.

a Adjusted for benign breast disease (0, yes; 1, no) and menopausal age (0<55 years; 1.55+ years).

).

The risk of being diagnosed with a hormone receptor-positive breast cancer following current use of HRT was significantly increased with an RR of 3.29 (2.27–4.77) compared to the never/past use. The risk of being diagnosed with the prognostic less favourable hormone receptor-negative breast cancer was not increased with the use of HRT; RR 0.99 (0.42–2.36). The difference between these two risk estimates among users of HRT was highly statistically significant (*P*=0.013) (Table 3a

**Table 3:** (a–c) The risk of invasive ductal carcinoma, with different prognostic characteristics following the use of HRT

**(a)**								
	**No of cases**	**Receptor status positive**	**No of cases**	**Receptor status negative**	**No of cases**	**Low malignancy grade (grade 1)**	**No. of cases**	**High malignancy grade (grade 2+3)**
**HRT use**	***N*=126/10 874**	**RR (95% CI)^a^**	***N*=32/10 874**	**RR (95% CI)^b^**	***N*=60/10 874**	**RR (95% CI)^c^**	***N*=101/10 874**	**RR (95% CI)^c^**
Never/past	63	1.00	25	1.00	26	1.00	*63*	1.00
Current	63	3.29 (2.27–4.77)	7	0.99 (0.42–2.36)	34	4.13 (2.43–7.01)	*38*	2.17 (1.42–3.30)
Current users pos *vs* neg receptor.status: *P*=0.013					Current users low *vs* high malignancy grade: *P*=0.063			
Never	54	1.00	19	1.00	22	1.00	*52*	1.00
Past	9	0.65 (0.32–1.31)	6	1.30 (0.48–3.55)	4	0.70 (0.24–2.03)	*11*	0.82 (0.43–1.58)
Current oestrogen	11	2.52 (1.31–4.84)	1	0.70 (0.09–5.22)	2	1.07 (0.25–4.56)	*10*	2.49 (1.26–4.91)
E+ prog-P Cyclical	15	4.78 (2.60–8.79)	0	na	10	7.62 (3.50–16.57)	*4*	1.50 (0.53–4.22)
E+ test-P Cyclical	19	2.63 (1.52–4.55)	4	1.62 (0.53–4.95)	9	2.85 (1.28–6.35)	*15*	2.27 (1.25–4.13)
E+ test-P Continuous	12	4.28 (2.22–8.24)	2	2.27 (0.52–9.90)	9	7.87 (3.59–17.28)	*6*	2.51 (1.07–5.87)
Current Tibolone	2	3.22 (0.78–13.25)	0	na	1	3.78 (0.51–28.10)	*2*	3.45 (0.84–14.21)
Current other HRT	4	1.86 (0.67–5.15)	0	na	3	3.34 (0.99–11.23)	*1*	0.51 (0.07–3.67)

**(b)**								
	**No. of cases**	**Lymph node status negative**	**No. of cases**	**Lymph node status positive**	**No. of cases**	**Small tumour size (<*2* cm)**	**No. of cases**	**Large tumour size (>2 cm)**
**HRT use**	***N*=93/10 874**	**RR (95% CI)^d^**	***N*=72/10 874**	**RR (95% CI)^e^**	***N*=97/10 874**	**RR (95% CI)^b^**	***N*=70/10 874**	**RR (95% CI)^c^**
Never/past	48	1.00	41	1.00	52	1.00	40	1.00
Current	45	2.58 (1.67–4.00)	31	2.52 (1.32–4.83)	45	2.57 (1.67–3.97)	30	2.74 (1.67–4.50)
Current users neg *vs* pos lymph node status: *P*=0.95					Current users small *vs* large tumour size: *P*=0.86			
Never	37	1.00	37	1.00	44	1.00	32	1.00
Past	11	1.11 (0.54–2.22)	4	0.26 (0.04–1.99)	8	0.83 (0.39–1.77)	8	0.95 (0.44–2.06)
Current oestrogen	6	1.86 (0.78–4.42)	5	1.89 (0.55–6.47)	5	1.47 (0.58–3.74)	6	2.41 (1.00–5.78)
E+ prog-P cyclical	8	3.03 (1.33–6.92)	7	3.11 (1.13–8.55)	8	2.77 (1.22–6.27)	7	4.40 (1.88–10.29)
E+ test-P cyclical	16	2.48 (1.31–4.68)	9	1.54 (0.60–3.98)	17	2.32 (1.23–4.36)	8	2.02 (0.90–4.51)
E+ test-P continuous	9	4.02 (1.86–8.70)	8	3.69 (1.24–10.98)	7	3.42 (1.53–7.68)	9	6.24 (2.94–13.22)
Current Tibolone	3	6.50 (1.99–21.18)	1	4.13 (0.55–31.17)	4	8.50 (3.03–23.82)	0	Na
Current other HRT	3	1.31 (0.31–5.45)	1	1.26 (0.17–9.52)	4	1.91 (0.59–6.21)	0	Na

**(c)**								
	**No. of cases**	**Low stage (stage 1)**	**No. of cases**	**High stage (stage 2–4)**	**No. of cases**	**NPI good prognosis**	**No. of cases**	**NPI moderate/poor prognosis**
**HRT use**	***N*=65/10 874**	**RR (95% CI)^d^**	***N*=97/10 874**	**RR (95% CI)^c^**	***N*=60/10 874**	**RR (95% CI)^b^**	***N*=92/10 874**	**RR (95% CI)^c^**
Never/Past	33	1.00	55	1.00	30	1.00	54	1.00
Current	32	2.49 (1.49–4.18)	42	2.71 (1.77–4.13)	30	2.61 (1.52–4.47)	38	2.48 (1.60–3.84)
Current users low stage *vs* high stage: *P*=0.81					Current users good prognosis *vs* mod./poor: *P*=0.89			
Never	26	1.00	47	1.00	24	1.00	46	1.00
Past	7	1.08 (0.47–2.52)	8	0.65 (0.31–1.38)	6	1.11 (0.45–2.74)	8	0.67 (0.31–1.41)
Current oestrogen	3	1.27 (0.38–4.22)	7	1.88 (0.85–4.18)	1	0.50 (0.07–3.70)	9	2.45 (1.20–5.03)
E+ prog-P cyclical	6	2.86 (1.077.57)	9	3.68 (1.76–7.71)	7	4.56 (1.93–10.78)	7	2.88 (1.27–6.52)
E+ test-P cyclical	12	2.54 (1.23–5.26)	13	2.17 (1.14–4.11)	12	2.47 (1.13–5.41)	11	1.83 (0.92–3.62)
E+ test-P continuous	5	3.36 (1.28–8.80)	11	5.11 (2.62–9.94)	5	3.90 (1.48–10.28)	9	4.29 (2.08–8.84)
Current Tibolone	3	8.61 (2.59–28.61)	1	1.93 (0.27–14.04)	2	7.03 (1.65–29.85)	1	1.97 (0.27–14.32)
Current other HRT	3	1.76 (0.42–7.45)	1	0.56 (0.08–4.10)	3	2.13 (0.50–9.05)	1	0.57 (0.08–4.14)

E=oestrogen, P=progestin, prog-P= progesterone-like progestin, test-P=testosterone-like progestin. NPI= Nottingham Prognostic Index.

a Adjusted for age, benign breast disease (0, yes; 1, no).

b Adjusted for age, age at menopause.

c Age adjusted.

d Adjusted for age, age at menopause (0<55 years; 1, 55+years) and use of oral contraceptives (0, no; 1, yes).

e Adjusted for age and night work (0, no; 1, yes).

).

The risk of being diagnosed with a breast cancer of low histological malignancy grade was increased following the current use of HRT compared to the never/past use, with an RR of 4.13 (2.43–7.01). This was nearly twice the risk of developing a breast cancer with a high malignancy grade, RR 2.17 (1.42–3.30), but in the model of competing risks this difference was not statistically significant (*P*=0.063).

Both the risk of being diagnosed with a lymph node-negative breast cancer and a lymph node-positive breast cancer was increased 2.5-fold in current users of HRT compared with never/past use. The risk of being diagnosed with a small tumour was equal to the risk of being diagnosed with a larger tumour of more than 2 cm. The RR estimates were equally increased for low- and high-stage breast cancer among users of HRT and the RR estimates of developing a good prognostic breast cancer or moderate/poor prognostic breast cancer according to NPI were equally increased as much as 2.5-fold (Table 3b and c).

The different HRT types and regimens all increased the RR of the various breast cancer types. The use of oestrogen only therapy tended to produce lower risk estimates in general, with no significant difference between the favourable and non-favourable breast cancer types, except for receptor status, where the highest estimate was seen for receptor-positive breast cancer. The risk estimates following the use of the continuous combined oestrogen–progestin regimens were generally higher than following the cyclical combined regimens, but with overlap in confidence intervals. The RR estimates with the use of progesterone-like progestins were higher than with the testosterone-like progestins when used cyclically, but numbers were small and confidence intervals overlap, indicating no difference. Tibolone was associated with the highest risk estimates, especially for the favourable breast cancer types, such as negative lymph node status, low stage at diagnosis and good prognostic group according to NPI. These risk estimates, however, were based on a small number of cases, allowing no further statistical comparison (Table 3).

## DISCUSSION

In the present study, we found that the current use of HRT increased the RR of being diagnosed with both favourable as well as nonfavourable breast cancer types, except for hormone receptor status, where we found significantly higher RR of hormone receptor-positive breast cancer compared to hormone receptor-negative breast cancer among current users of HRT. We found a higher risk of being diagnosed with histological low malignancy grade as compared to high malignancy grade in current users of HRT, but this difference was not statistically significant.

Histological malignancy grade and hormone receptor status represent biological variables, whereas the other prognostic factors represent time-dependent variables, such as tumour size, lymph node status and TNM stage. As 90% of breast tumours with low malignancy grade (grade 1) are hormone receptor positive, the two prognostic factors somehow resemble each other. A total of 48% of the current HRT users had tumours that were both hormone receptor positive and had low malignancy grade, as opposed to 27% among never users of HRT. Current HRT users diagnosed with tumours of both high histological malignancy grade and negative receptor status comprised 8%, while the corresponding figure for never users was 25%. These rough comparisons indicate that the effect seen for the histological malignancy grade probably is to be explained by receptor status. However, our data do not allow for further statistical comparisons or stratified analyses as numbers are too small and thus additional follow-up time is required.

**Figure 1:**
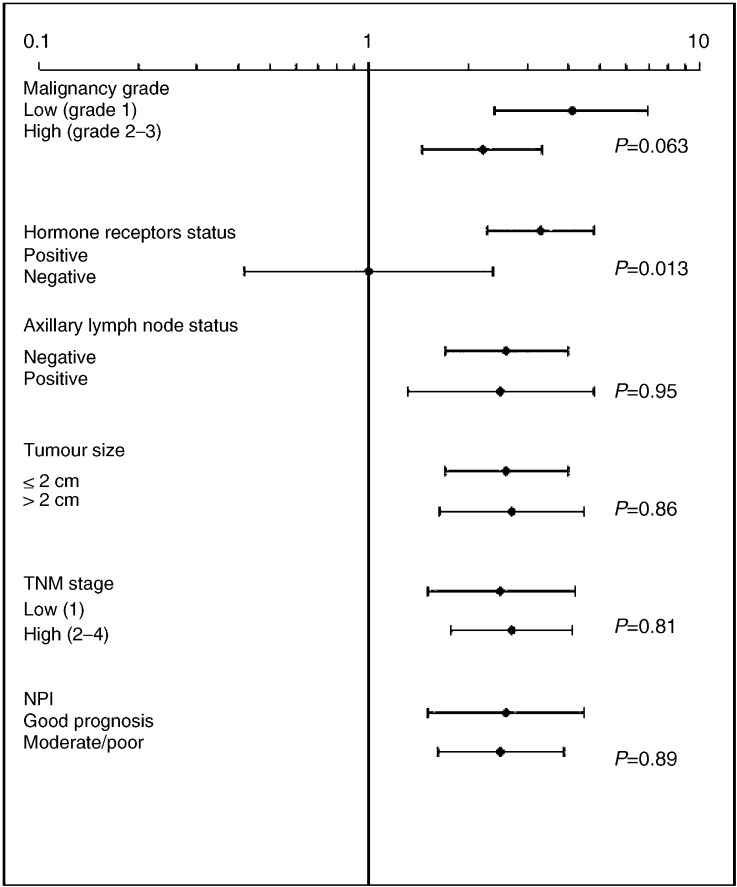
Risk of breast cancer with different prognostic characteristics in current users of HRT compared to never users, with 95% confidence intervals.

For the other prognostic factors being time-dependent and reflecting the stage and development of the disease, such as lymph node status and tumour size, the risk estimates for the favourable and non-favourable outcomes were equally increased among current users of HRT and past/never users. NPI including both the biological and time-dependent variables showed equally increased risk estimates throughout prognostic groups (Figure 1

).

Our study does not support the notion that breast cancer in users of HRT is of larger size or diagnosed at a more advanced stage, nor does our study indicate earlier diagnosis with smaller tumours and less advanced stage of disease as supposed by the possible influence of increased surveillance among users of HRT.

Most previous studies have compared the distribution of prognostic factors in breast cancer cases only among users of HRT and non-users, respectively. If, in our study, we merely had focused on the favourable prognostic factors in breast cancer cases, we would have found a RR of 1.46 (0.99–2.17) for developing a hormone receptor-positive breast cancer among users of HRT. These estimates do not reflect the overall increased incidence of breast cancer with the use of HRT, nor do they reflect the risk of developing the unfavourable type of breast cancer, and thus might be misleading. Hence, it is important to base the analysis on the entire study population, analysing both the favourable and nonfavourable types of breast cancer according to HRT.

In the present study, we have furthermore tested whether there was a difference in being diagnosed with a more favourable cancer compared to a nonfavourable cancer in current users of HRT, by a model of competing risks analyses. Similar to our analytic approach, a recent study has analysed the risk of being diagnosed with various types of breast cancer in women attending mammographic screening. The risk of being diagnosed with both favourable and nonfavourable types of breast cancer was increased for users of the combined oestrogen–progestin regimens. Even if no analysis of competing risks was performed, the confidence intervals outline a higher risk of developing an oestrogen receptor-positive rather than a receptor-negative breast cancer in users of HRT for 5 years or more. Furthermore, users of HRT had smaller tumours at the time of diagnosis, which is counterbalanced by a higher rate of false-negative examination results (Kerlikowske *et al*, 2003).

It has previously been shown that in this study population there is an increased risk of breast cancer following the use of HRT and that this risk is more pronounced for the combined oestrogen–progestin HRT regimens, especially for the continuous combined HRT regimens (Stahlberg *et al*, 2004). The estimates in the present analysis seem to confirm these findings; however, numbers are smaller because of the subdivision into various breast cancer types of different prognostic characteristics. The risk of breast cancer following the use of tibolone was significantly increased for all types of breast cancers, most so for some favourable types, but the estimates are based on small numbers and confidence intervals are wide. This finding is partly unexpected, as early reports have suggested a beneficial effect on breast tissue with the use of tibolone and the mammographic density was unaffected (Chetrite *et al*, 1999; Lundstrom *et al*, 2002). Tibolone might therefore have been preferred for women with a family history of breast cancer, as it has been considered a safer HRT regimen regarding the risk of breast cancer. It has, however, been proposed in an *in vitro* study that tibolone does have tumour cell-growth-promoting effects and therefore might be unsuited for women at high risk of breast cancer (Lippert *et al*, 2002). As our data on family history of breast cancer are limited, we have not been able to control for familiar predisposition in our analyses.

Few other studies have analysed breast cancer prognostic characteristics according to different HRT types and regimen (Magnusson *et al*, 1996; Ross *et al*, 2000; Schairer *et al*, 2000; Daling *et al*, 2003). A Swedish study found that combined oestrogen and progestin use was associated with lower risks of being diagnosed with larger tumours above 2 cm odds ratio (OR) 0.3 (0.1–0.7) and with positive axillary lymph nodes OR 0.7 (0.4–1.1) compared to never users. A study from USA found that oestrogen-only therapy was associated primarily with CIS lesions, while the combined oestrogen and progestin treatment was associated with an increased risk of both *in situ* lesions as well as low- and high-stage invasive breast cancer (Ross *et al*, 2000). Recent studies have found that oestrogen-only users had decreased odds of being diagnosed with distant disease and that tumours in women having used the combined oestrogen–progestin HRT regimens were more often both oestrogen- and progesterone-receptor positive (Daling *et al*, 2003; Li *et al*, 2003). There is no agreement as to whether the estimates are highest for the continuous or the cyclically combined regimens. The recent study by Kerlikowske *et al* used proxy-variables for HRT type, as it was assumed that women with an intact uterus had used combined oestrogen–progestin regimens and women without a uterus had used oestrogen-only therapy regimens. This might, however, introduce bias when analysing the effect of different HRT regimens.

Observational studies have certain limitations and strengths. As this is a prospective study, there is no recall bias active with respect to exposure information on HRT use. This study comprises a nationwide cohort of Danish nurses and bias by education/social class is therefore not likely to affect the results. The information on HRT types and regimens was available only for current users at baseline, and as in many other prospective cohorts we have no information on updated exposure. Therefore, misclassification might occur over time. However, it has previously been shown that there was a high degree of adherence to therapy regimens among Danish women using HRT, especially with the use of the continuous combined regimens over time (Eiken and Kolthoff, 2002). Our data on familiar predisposition are limited and thus in this study we could not control for this potential confounding factor. A strong familiar and genetic predisposition would often result in early premenopausal onset of breast cancer, and breast cancers developing in younger women have been associated with poor prognostic characteristics such as high histological malignancy grade (Fisher *et al*, 1997; Kollias *et al*, 1997). However, this cohort comprises of postmenopausal women only and women with previous breast cancer have been excluded from analysis. Furthermore, previous studies have not found any effect modification by familiar predisposition on the use of HRT (Ursin *et al*, 2002). Surveillance or detection bias is a major source of bias when analysing the risk of developing breast cancer with different prognostic characteristics in women using HRT. We have no information on the mode of detection in our study. In Denmark, only two out of 15 counties had mammographic screening established during the time of observation. The radiographic detection of breast cancers in women using HRT might be more difficult because of more dense mammograms, resulting in a higher false-negative rate in mammographic screening rounds (Chlebowski *et al*, 2003; Kerlikowske *et al*, 2003). On the other hand, both the physician prescribing HRT and the woman herself will probably be more aware of the risk of cancer development. This may result in higher rates of both referrals from physicians and HRT users for mammographic and ultrasound examination, even in a screening population. Nurses may have easier access to radiographic examination as self-referrals. A minor detection bias is therefore a possibility. However, we compared the stage distribution in our study with the national results from the DBCG registry, where low-stage breast cancer during the period of follow-up was defined as negative lymph node status and tumour size less than 5 cm. The national results from the treatment protocol of 1989 showed that 57% of all breast cancers were low-stage tumours, which compares to the 55.6% in the present study. The stage distribution is thus the same, indicating that breast cancer diagnosed in Danish nurses resembles the whole female population. The higher risk of developing breast cancer with a positive receptor status and to some extent low histological malignancy grade is therefore considered likely to be associated with the prior treatment with HRT.

## CONCLUSION

In conclusion, the current use of HRT increases the risk of being diagnosed with both favourable and nonfavourable breast cancers two- to four-fold. This study shows that, in current users of HRT at baseline, the RR of developing breast cancer with positive hormone receptor status is increased, while the risk of developing a hormone receptor-negative tumour is neutral as compared to the never/past use. This difference is statistically significant. For the other prognostic factors, we find no significant difference in risk estimates for the favourable and nonfavourable prognostic characteristics with the current use of HRT. Concerning different HRT regimens, the risk of breast cancer is increased following the use of any type of HRT, but the highest estimates are seen with the use of the continuous combined oestrogen and progestin HRT regimen.
